# Performance Evaluation of UF Membranes Derived from Recycled RO Membrane, a Step towards Circular Economy in Desalination

**DOI:** 10.3390/membranes13070628

**Published:** 2023-06-28

**Authors:** Zia Ur Rehman, Hira Amjad, Sher Jamal Khan, Maria Yasmeen, Aftab Ahmad Khan, Noman Khalid Khanzada

**Affiliations:** 1 Institute of Environmental Sciences and Engineering IESE, National University of Sciences and Technology, NUST, Islamabad 24090, Pakistanmariarajpoot919@gmail.com (M.Y.); 2 Department of Civil and Environmental Engineering, Hanyang University, Seoul, 04763, Republic of Korea; 3 Creative Engineering Consultants, Peshawar 25100, Pakistan; 4 School of Energy and Environment (SEE), City University of Hong Kong, Hong Kong SAR, China

**Keywords:** spent reverse osmosis (RO) membrane, spent RO recycling, integrated constructed wetland (ICW), membrane bio-rector permeate (MBR), circular economy

## Abstract

Reverse osmosis (RO) spiral wound membrane generation reached 93.5% in 2020, resulting in 14,000 tons of used RO membranes being discarded annually into landfills, which is unprecedented. The current study aims to chemically convert the end-of-life RO membrane, followed by its performance evaluation and microbial removal efficiency on three different sources of water, i.e., tap water (TW), integrated constructed wetland permeate (ICW-P), and membrane bio-rector permeate (MBR-P), respectively. This was accomplished by selecting 6 years of spent Filmtech (LC-LE-4040) thin film composite type brackish water reverse osmosis (BWRO) membrane, followed by alkaline and acidic cleaning for 2 h. Finally, the conversion was carried out by 6% sodium hypochlorite (NaOCl) with 300,000 ppm/h exposure by active system (AS) using the clean in place CIP pump at 2 bars for 10 h duration. The membrane demonstrated 67% water recovery and 1% saltwater rejection, which means RO membrane now converted into recycled RO (R-RO) or (UF) by removal of the polyamide (PA) layer. Water recovery was 67% for TW, 68% for ICW-P, and 74% for MBR-P, respectively, with the consistent saltwater rejection rate of 1% being observed, while R-RO exhibited an effective COD removal of 65.79%, 62.96%, and 67.72% in TW, ICW-P, and MBR-P, respectively. The highest turbidity removal of 96% in the ICW-P was also recorded for R-RO. For morphological properties, SEM analysis of the R-RO membrane revealed a likewise appearance as a UF membrane, while pore size is also comparable with the UF membrane. The most probable number (MPN) also showed complete removal of total coliforms after passing through the R-RO membrane. These features made the R-RO membrane an excellent choice for drinking water treatment and wastewater treatment polishing steps. This solution can help developing nations to be efficient in resource recovery and contribute to the circular economy.

## 1. Introduction

Desalination is an ancient technique to obtain clean water. With rapid population growth, the demand for water has increased. At present, water on the earth is accounted for with 97% as oceanic/salty, and the rest (3%) is freshwater which creates a great deal of interest in supplying clean water to humanity [1,2]. Historically on ships, distillation was a process where heat was used to separate water from salt, which was later used and developed for voyages in the late 18th century. The history of desalination started when commercial desalination plants were being deployed (1881–1907) and were installed in Tinge, Malta, and Jeddah, Saudi Arabia, respectively [3,4]. In the past, it was conducted in the form of thermal distillation techniques such as multi-flash distillation and multiple effect distillation, installed foremost in the Middle East. Later on, this was shifted towards desalination due to the high footprint of energy consumption and the emergence of the RO membrane [5,6]. The reverse osmosis (RO) membrane was a revolutionary advancement in the desalination sector [7], especially the spiral wound membrane discovered by Westmoreland, Bray, in the late 1960s [8]. It is a rolled configuration with enhanced surface area, which sustains more pressure and exhibits higher saltwater rejection % and water recovery %. The capacity of RO membrane use has increased as compared to Multi-Stage Flash (MSF) and Multiple-Effect Distillation (MED) and is expected to reach production of 6 million m^3^/day, according to the data from the Global Water Intelligence(GWI)/Desal 2020 report [9,10].

The (Thin-Film Composite) TFC polyamide RO membrane is most abundantly used in the world and has large footprint for desalination, and its fouling leads to a reduction of lifespan of 5 to 10 years which ultimately goes towards the landfill and creates more waste to deal with [11,12].

Due to unsustainable consumption patterns mixed with rising industrialization, urbanization, and population growth, the environment and natural resources are under stress. Environmental issues such as climate change, air pollution, biodiversity loss, and the degradation of water and soil are often caused by economic development and underlying patterns of unsustainable production and consumption [13]. The circular economy addresses decoupling, resource efficiency, production efficiency, slower material flows rather than linear economic models, and decreased resource extraction without diminishing economic activity [14].

In the linear economy, things are made, used, and disposed of while in the circular economy the resource is recycled instead of going to waste, its life is enhanced, and this poses less threat to the environment [15]. Using these spent RO membranes to convert them into Ultrafiltration (UF) membranes is one such approach that can significantly contribute in the circular economy to deal with the 2 million expected spiral-wound spent RO membranes by 2025 [16,17].

The RO membrane, by removing the top layer of PA, can be converted into a UF membrane with higher permeability, and excellent performance can be achieved for many purposes [18]. Jawad in 2021 converted the RO membrane and tested it on the gray water that showed higher water recovery % and removal of *E. coli* [19]. Khaless et al. in 2021 observed the salt rejection percentage of the RO spent membrane and proposed to use it for phosphoric acid treatment [20,21]. Salinas et al. in 2020 evaluated the life cycle assessment (LCA) and direct economic analysis of recycling of RO membranes that showed positive results, and it proved effective as an environmental benefit, and recycled modules can be sold at a competitive price of 80 euros in the market [22,23].

The Pakistan Council of Research in Water Resources (PCRWR) has recently published a report on the drinking water quality in Pakistan and its current status and challenges in 2021, which was conducted in 29 cities of 4 provinces, revealing that out of 435 water sources, when compared with National Standards for Drinking Water Quality Standards (NSDWQ), 267 (61%) sources showed that they are unsafe for drinking. There were 11 major issues including 41% being microbiological issues, turbidity (9%), nitrates (4%), pH (1%), and others [24]. Pakistan and other developing countries also need membrane (RO/UF pilot scale plants) solutions that must be cost-effective, sustainable, and efficient in performance to provide pure and clean drinking water to its people, but the cost is an eminent factor that curtails the achievement of this objective [25,26,27].

The novelty of study lies in the evaluation of recovered RO membranes as tertiary treatment for integrated constructed wetland permeate and membrane bioreactor (MBR) permeate, which have not been previously investigated. Furthermore, the performance of this recovered membrane was compared with drinking water standards. This unique approach allows us to assess the suitability and effectiveness of the recovered membranes in reusing water from different types of sources. This research explored diverse water and wastewater permeate treatment scenarios, expanding the possibilities for sustainable and cost-effective water purification solutions. This study aims (1) for the chemical conversion of spent RO membrane into recycled RO or ultrafiltration for water treatment and (2) to observe the performance and microbial removal efficiency on different sources of water, i.e., tap water (TW), integrated constructed wetland permeate (ICW-P), and membrane bio-rector permeate (MBR-P).

## 2. Material and Methods

### 2.1. RO Pilot Plant Description

This study used the pilot scale (RO plant), with a brackish water reverse osmosis (BWRO) membrane embedded from the past 6 years, previously used for synthetic brackish water evaluation and a performance study conducted by Khanzada in 2017 [28].

**Figure 1:**
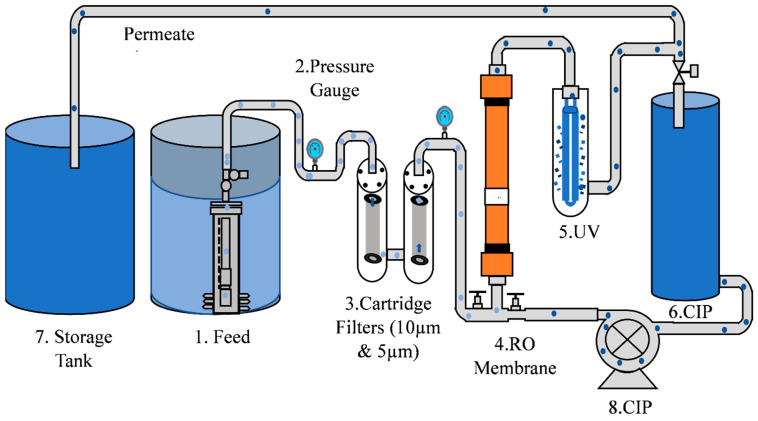
Process flow diagram (1) Feed tank, (2) Submersible feed pump, (3) Cartridge filters 10 and 5 µm, (4) RO module, (5) Ultraviolet sterilizer (UV), (6) CIP tank, (7) Storage tank, and (8) CIP pump.

**Figure 2:**
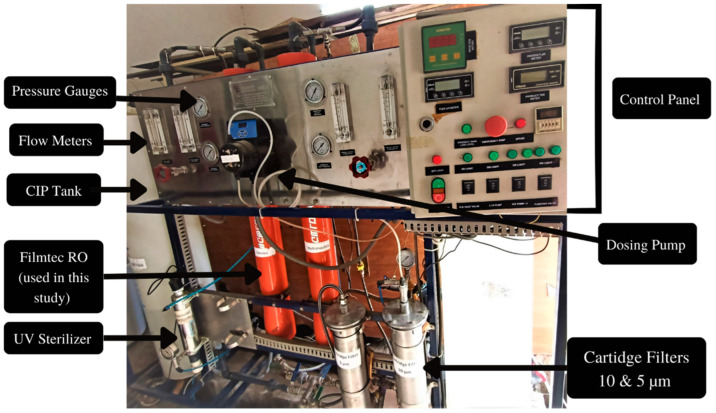
Pilot scale reverse osmosis experimental setup installed at NUST.

The system as shown in Figure 1 comprises a submersible feed pump (Model: SQF 0.6–3, Grundfos, Edenbridge, UK) placed inside the feed tank of 200 US gallon (757 L) water capacity, followed by 2 melt blown cartridge filters (CF), 10 µm and 5 µm, respectively, an end-of-life RO membrane thin film-composite TFC type (Model: Filmtech LC-LE-4040) of size 4 by 40″, an ultraviolet sterilizer UV lamp (Model: Wonder Light Stainless Steel PC-2: 2 GPM 110v), and a clean in-place pump CIP (Model: MSP 230, Marchmay, Saint Neots, UK) and a CIP tank as well, while the outlet and inlet membrane pressure were measured by pressure gauges (model: 233.55 LBM, WIKA Instrument Corporation, Lawrenceville, GA, USA), a continuous meter for TDS (model: TDS consistent monitor 230), and a pH meter placed in the plant. The actual pilot scale experimental setup is shown in Figure 2.

### 2.2. Operational Details and Parameters

The BWRO membrane is a polyamide thin-film composite TFC-type spiral wound membrane used in the study. In the composition, the RO membrane polyamide (PA) is at the top, followed by polysulfone (PSF) and a base of polyester.

The PA layer is highly susceptible to chlorine exposure and starts degrading at 1000 ppm/h due to the deformation in its structure as Singh explained in 2006. Membrane properties are described in Table 1.

The recycled RO (R-RO) membrane water recovery %, saltwater rejection %, permeability, and transmembrane TMP are calculated during the operation.

For water recovery percentage following Equation (1) was used [29,30].

(1) $$\mathrm{Water}\;\mathrm{recovery}\;\%=\frac{Q_{p}}{Q_{f}}\times 100$$

where Q*_p_* is permeate and Q*_f_* is feed water flow in (L/h).

Saltwater rejection % was determined using Equation (2) [30].

(2) $$\mathrm{Saltwater}\;\mathrm{rejection}\;\%=\left(1-\frac{C_{p}}{C_{f}}\right)\times 100$$

where C*_p_* refers to permeate and C*_f_* refers to feed concentrations, respectively.

### 2.3. Conversion Procedure

When the spent RO membrane is treated with 300,000 ppm conc. per hour of sodium hypochlorite, it removes the top layer of PA by converting the membrane into recycled RO (R-RO) or UF membrane. This procedure is well-documented and performed as followed by [20] that sodium hypochlorite exposure degrades the polyamide layer with the above concentration.

For conversion, an active system (AS) followed in which, at the start, alkaline cleaning was performed using 0.1% of sodium hydroxide (NaOH) in pellet form purchased from Sigma Aldrich (Burlington, MA, United States) dissolved in 14 L of distilled water and stirred for 5 min [31]. The exposure time was 2 h, at 2 bar pressure, and the CIP pump was used for the alkaline cleaning process, and 5–10 min relaxation time was given after one hour. Acidic cleaning using 2% Citric Acid (C_6_H_8_O_7_) procured from Sigma Aldrich was followed with the same method as for the alkaline cleaning [32,33,34].

Some 6% sodium hypochlorite (NaOCl), i.e., 1200 mL of 12.5% concentration, was purchased from VWR Chemicals International, dissolved in distilled water (20 L), stirred for 5 min, and operated at 2 bar pressure for the 5 h of exposure time, but the required result was not achieved after the first process. So, the whole conversion with all the same details has been repeated one more time. The same concentration of NaOCl was applied a second time, and a net total of 2400 mL was used to convert the spent RO into an R-RO membrane [21]. The conversion procedure details are given in Table 2.

### 2.4. Characteristics of Water

Realizing the potential of the R-RO (Recycled RO) membrane, it was tested on the 3 different sources of water, i.e., tap water (TW) near the constructed wetland with a 16 m distance, the second type of membrane bio-rector permeate (MBR-P) water, and constructed wetland permeate (ICW-P)-treated water.

All the initial findings of the parameters conducted are compiled in Table 3.

Initial physicochemical and microbial analysis of TW, ICW-P, and MBR-P was measured in laboratory using pH meter (Model: Bench-top pH meter HI 8520 microprocessor); electrical conductivity was measured by an EC meter (Model: conductivity meter Cond 720); and turbidity was measured using a portable turbidimeter (Model: HACH 2100P). For chemical oxygen demand (COD), the media prepared using potassium dichromate (K_2_Cr_2_O_7_) purchased from Duksan International. Sulfuric acid (H_2_SO_4_) was purchased from Sigma Aldrich, and then after the addition of the sample, it was digested for 2 h. The sample was titrated against ferrous ammonium sulphate (NH_4_)_2_Fe(SO_4_)_2_·6H_2_O obtained from Duksan International, until a reddish brown color appeared.

The most probable number (MPN) was performed using 3 different media: lauryl tryptose broth LTB (code: CM0451), brilliant green bile BGB 2% (code: CM0031), and EC broth (code: CM0853), all procured from oxoid. For MPN, media were prepared, autoclaved for 2 h, and then placed for incubation at 37 °C overnight, and the sample was inoculated the next day followed by incubation at the same condition. On the second day, there was a check of the LTB tubes and a count of the positive numbers which are turbid, and the sample in BGB tubes was transferred using laminar flow, and the same procedure was repeated for the EC tubes as well, and then the number of positive tubes from the MPN index was checked.

### 2.5. Membrane Characterization

#### Scanning Electron Microscopy (SEM) Analysis

R-RO membrane samples were prepared and dried at 40 °C. Then, to analyze surface morphology of the membrane after chlorine exposure, SEM analysis was conducted using the SEM (model: MIRA3 TESCAN) from the Institute of Space Technology (IST), Islamabad. Then, pore size was determined using the same software. These high-resolution micrographs provide the results to evaluate the surface and were compared with the recycled RO membrane. Energy Dispersive X-ray Analysis (EDX) was also measured to know the concentration of different elements’ presence [35,36].

## 3. Results and Discussion

### 3.1. RO to R-RO (Degradation of Polyamide PA Layer)

**Figure 3:**
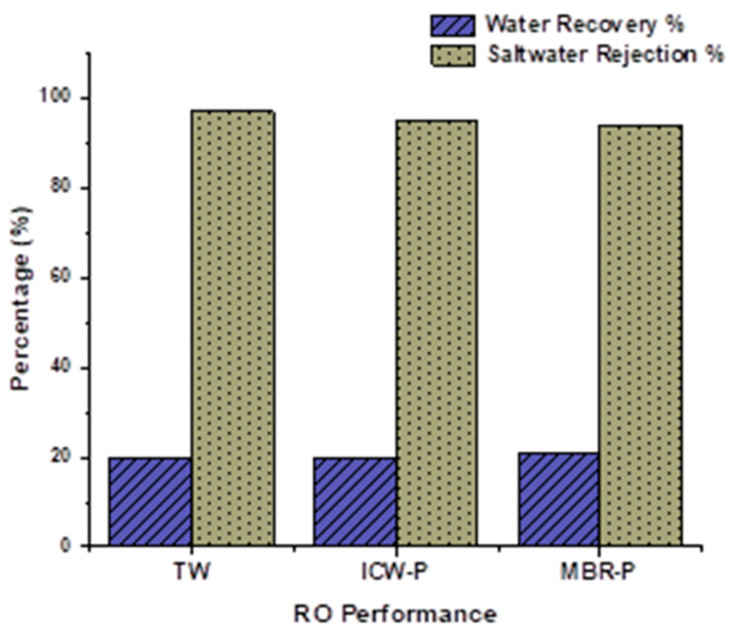
Performance of RO membrane before conversion.

The RO spent membrane showed a 20% water recovery percentage and a very high saltwater rejection percentage of 94%, 95%, and 96% on TW, ICW-P, and MBR-P, respectively, as shown in Figure 3 at the start of the study. After 6 years, the membrane still exhibited a considerable amount of saltwater rejection % but a low water recovery %.

**Figure 4:**
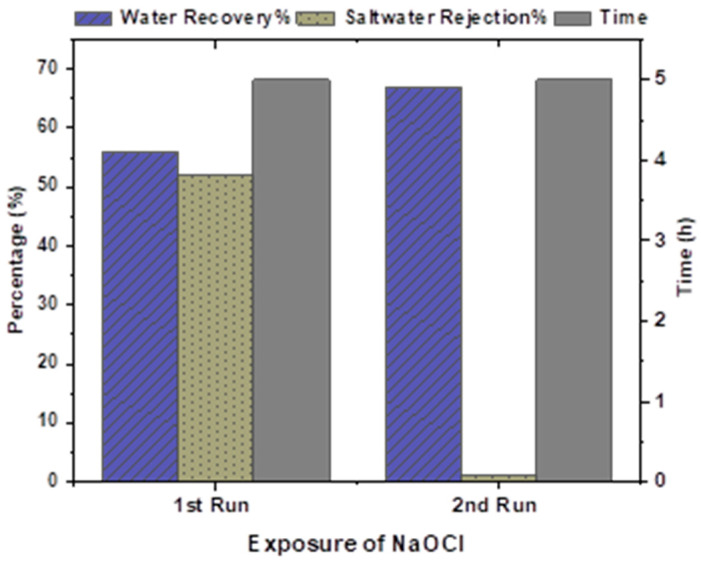
Exposure of NaOCl on the RO membrane, water recovery %, and saltwater rejection % with respect to time.

Figure 4 shows that upon the first run of the plant at 1200 mL of NaOCl, water recovery increased from 20 to 56% but the saltwater rejection decreased to 52% which means the still PA layer exists and needs more chlorine exposure to further degrade the layer and expose the polysulfone (PSF) layer. A similar study observed that low exposure of chlorine at 50–1000 ppm/h caused an increase in the membrane permeability without reducing the saltwater rejection % of the RO membrane, which was conducted by Garcia [37].

**Figure 5:**
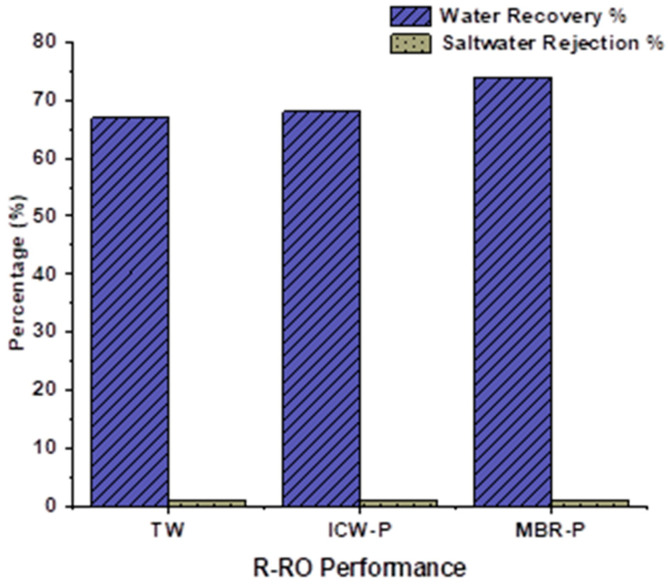
Performance of RO after conversion.

Then, after the second run, the saltwater rejection % was 1%, and water recovery increased up to 67% which means the PA layer had been degraded and the PSF had been exposed. Now, it is known as a Recycled RO membrane (R-RO), and it lies between virgin RO and the UF membrane because of its performance. The R-RO performance remained consistent on all sources of water in terms of rejection %, but an increase in the water recovery with time has been observed. It was 67% for TW, 68% for ICW-P, and 74% for MBR-P, respectively, as depicted in Figure 5.

Another study on UF spiral wounds was conducted by Mierzwa et al. using the Guarapiranga Reservoir, a eutrophicated body, as a source which revealed that virgin UF showed 85% water recovery and 95% removal of turbidity [38] which is comparable with the recycled RO that showed a maximum of 74% water recovery. It is further enhanced with proper cleaning and backwashing.

### 3.2. R-RO Permeate Flux and TMP

**Figure 6:**
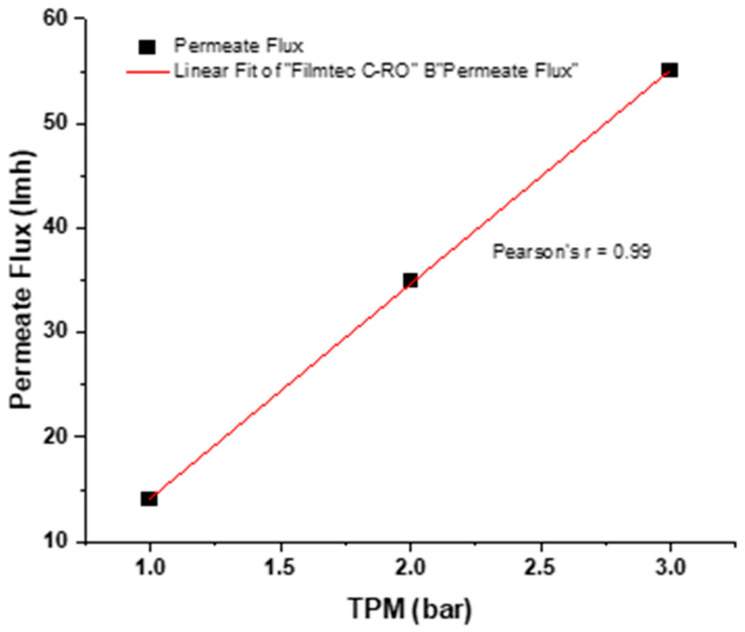
R-RO Permeate flux with respect to TMP.

With the increase in TMP, a higher permeate flux rate was observed. At 1 bar, permeate flux was 14 lmh; at 2 bars, it was 35 lmh; and at 3 bars, it spiked to 55 lmh, respectively, as shown in Figure 6. The higher permeability of R-RO can be attributed to the exposure of chlorine 300,000 ppm/h and complete degradation of the PA layer [39]. It was observed that of the various foulants the membrane encountered while operating, the efficiency of the cleaning and the conversion process all contributed to the difference in converted membrane permeability.

### 3.3. SEM Analysis of R-RO Membrane

**Figure 7:**
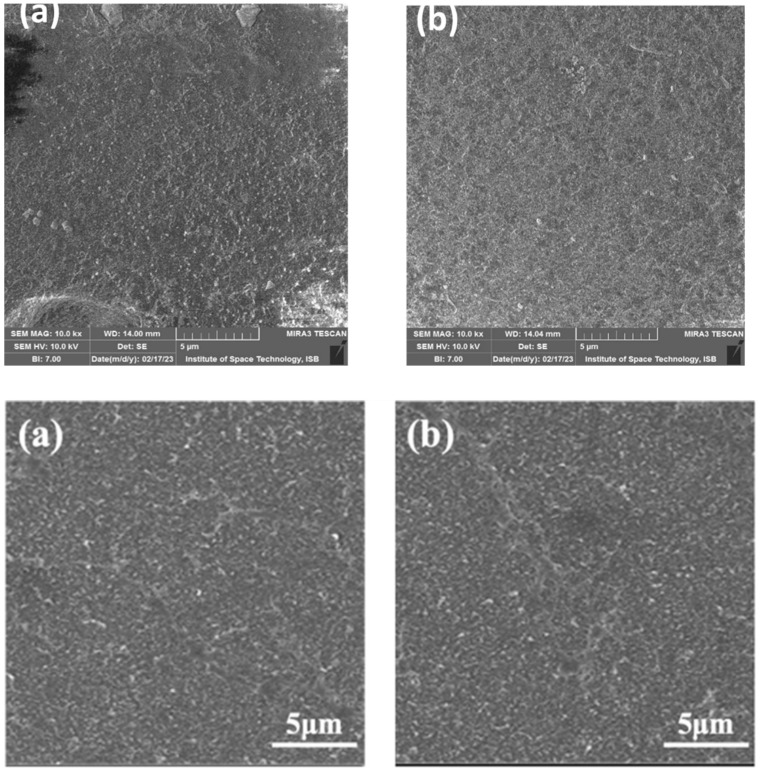
SEM micrographs at 5000 magnification, (**top a**,**top b**) recycled RO membrane after 10 h exposure at 5000 magnification, (**lower a**) SEM micrograph of R-RO after 0 h (**lower b**) after 12 h from the literature at the same magnification for comparison.

The micrograph of the recycled RO membrane in Figure 7 shows the removal of the polyamide layer and large pore sizes. After 10 h of exposure with sodium hypochlorite solution, the PA layer has been removed, similar to the membrane morphology presented in a study [31].

**Figure 8:**
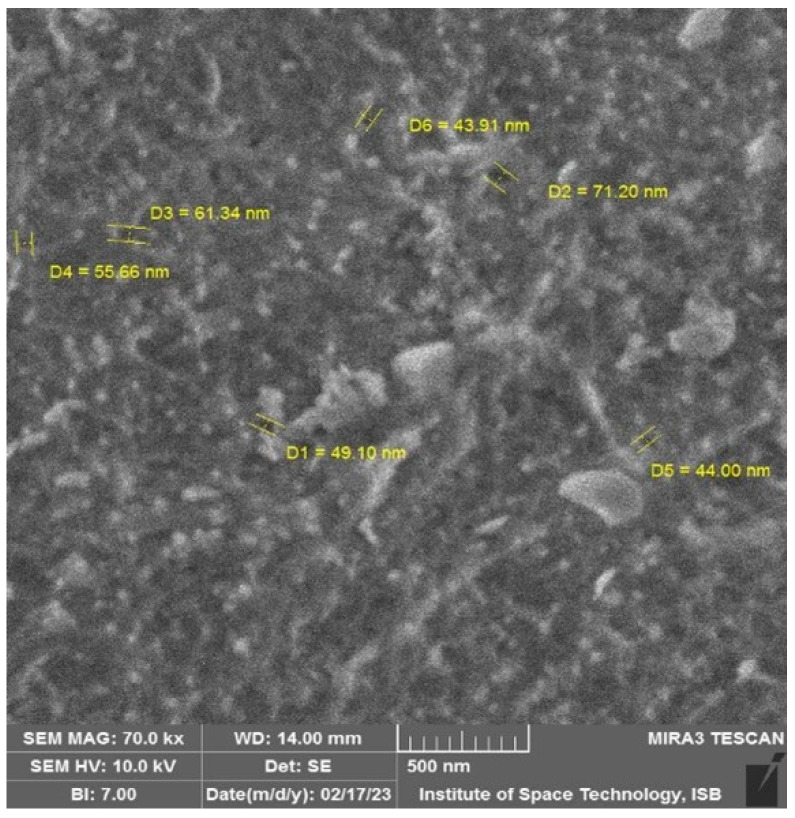
Pore size of the R-RO membrane at 500 nm magnification.

Figure 8 shows that the pore size range of R-RO membrane at 500 nm magnification is the same as UF membrane. Figures S3–S5 in supplementary information shows R-RO membrane at 1000 nm, 2000 nm and 500 nm respectively. One of the previous studies showed that UF membrane pore size usually ranges between 1 and 100 nm depending on the different brands of the membrane. Therefore, an end-of-life RO membrane has been converted into recycled RO or a converted UF membrane [40].

**Figure 9:**
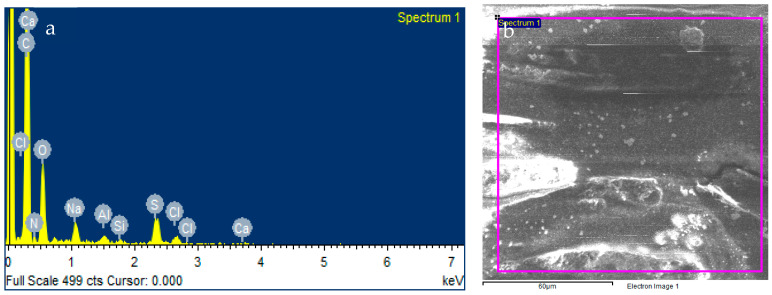
(**a**) EDX spectra of elements, (**b**) SEM micrograph.

While in Figure 9 the SEM-EDX analysis of the sample shows the weight percent and atomic percent of various elements present in it, the analysis reveals that the sample is primarily composed of carbon (C) and nitrogen (N), with weight percentages of 38.21% and 37.24%, respectively. Oxygen (O) is the third most abundant element, with a weight percentage of 22.21%.

Other elements present in the sample include sodium (Na), aluminum (Al), silicon (Si), sulfur (S), chlorine (Cl), and calcium (Ca), with weight percentages ranging from 0.02% to 0.87% in Table 4. The atomic percentages of these elements are also provided for reference. These results can provide insights into the composition and potential applications of the sample, which can be further investigated in future studies. These elements can be validated from previous research [41,42].

### 3.4. Physicochemical and Microbial Parameters

**Figure 10:**
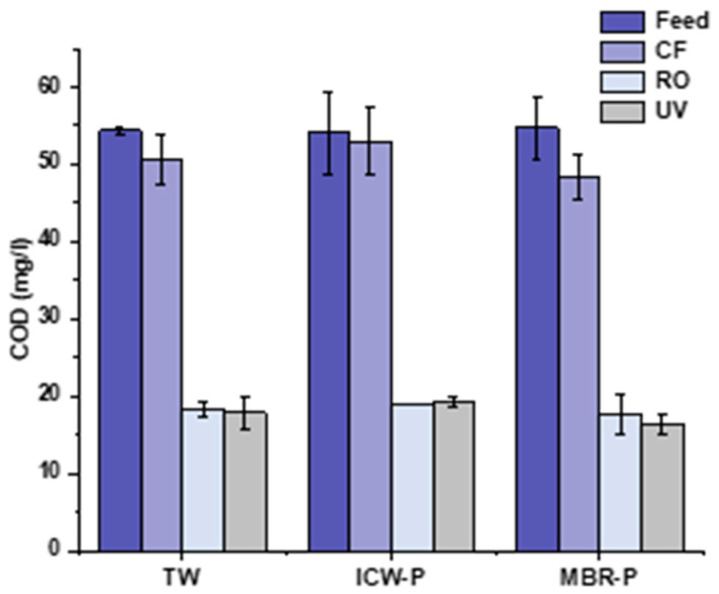
COD removal efficiency of the R-RO membrane in TW, ICW-P, and MBR-P waters.

The R-RO membrane showed an excellent removal of COD efficiency as shown in Figure 10. In the feed of tap water, COD was 52 mg/L, with a slight decrease after passing through the cartridge filter (CF) of 43 mg/L, of 18 mg/L for COD after passing through the recycled RO, and of 17.7 mg/L after the UV lamp. A 65.79% COD reduction has been observed after the R-RO membrane. In all three runs, the performance of the membrane remained consistent in the tap water, which reiterates a higher capacity to remove the contaminants sustained inside the membrane.

In ICW-P feed water, the COD was 54 mg/L, with a minor change after CF 52 mg/L, and was 20 mg/L after passing through the R-RO membrane with a 62.96% COD removal efficiency, and after a UV reading, 19 mg/L was observed.

In MBR-P water used as feed, the COD was recorded as 54 mg/L, followed by 48 mg/L, 17 mg/L, and 17 mg/L in CF, R-RO, and UV, respectively, with 67.72% COD removal efficiency after passing through the membrane. It is the highest COD percentage removal found among three sources of water used in the study. Sumisha et al. checked the ultrafiltration membrane on the laundry wastewater and its capability of COD reduction efficiency which showed that the polyethersulfone (PES) membrane achieved 88% COD removal with 10% added PVP, while the R-RO membrane has expressed a challenging COD percentage reduction across the sources of water operated on in contrast with virgin UF membrane [43].

Liu et al. found out that the virgin RO membrane showed a COD less than 10 mg/L being tested, and also the COD removal % rate varies from 90 to 94% [44] while the R-RO COD removal % reduction in comparison to virgin RO can be attributed to the PA layer depletion and more fouling with the time on the membrane surface.

### 3.5. Turbidity Removal %

**Figure 11:**
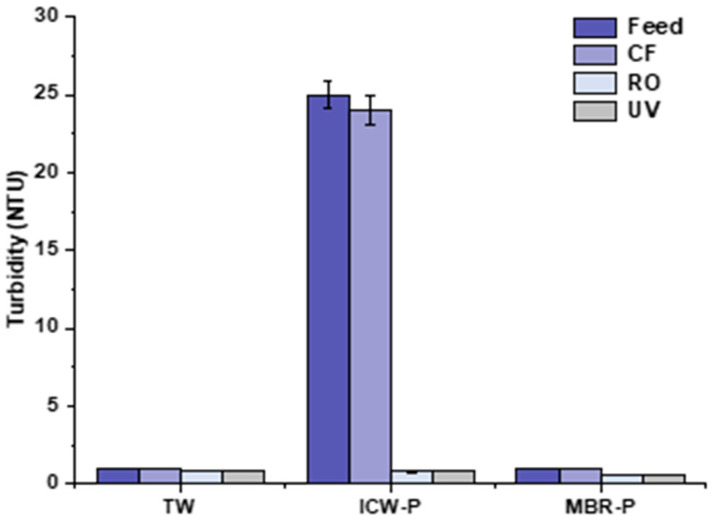
Turbidity removal efficiency of R-RO membrane in TW, ICW-P, and MBR-P water.

The turbidity removal is depicted in Figure 11. In the TW feed water, it was <1 NTU and remained less than 1 passing through CF, R-RO, and UV, respectively, while during the ICW-P run, the highest turbidity was observed as 25 NTU, followed by 24 NTU after the CF and 1 NTU after the R-RO membrane, and it remained the same after UV as well. The Filmtec R-RO demonstrated removal of 96% by removing all the suspended solids present in the integrated constructed wetland permeate (ICW-P). This performance of the recycled RO membrane is comparable with the virgin UF in terms of turbidity removal percentage, as Zulaikha et al. in the study reported UF (PES-10 kDa) used to treat the wastewater and demonstrated 99% turbidity removal while the Filmtec recycled RO membrane showed 96% removal. The efficacy of the recycled RO membrane is comparable with the virgin UF in terms of turbidity removal percentage [45].

### 3.6. Total Coliforms Removal %

The MPN results in Table 5 demonstrated the presence of total coliforms more than >23 CFU/100 mL with a 13-confidence limit in the feed. The second stage CF did not show any removal, while the R-RO membrane showed all negative tubes, and the index shows a confidence level of 3.4 and 100% total coliform removal in all three sources of TW, ICW-P, and MBR-P water.

The reason tap water results were similar is because of its source distance from the integrated constructed wetland ICW since its inception which shows evidence of groundwater contamination due to ICW operation. In the feed water of ICW-P and MBR-P, homogeneous results were found, and no microbial contamination was detected after R-RO membrane.

All water sources after passing through the recycled RO membrane were found to meet the WHO’s [46] standards and Pakistan’s National Standards for Drinking Water Quality (NSDWQ) limits [47]. In the case of tap water(TW) for drinking purposes and as wastewater effluent discharge for the integrated constructed wetland permeate (ICW-P) and membrane bio-rector permeate (MBR-P) is shown in Table 6.

The findings of the study emphasize that the resource (discarded RO membrane) has recovered, which contributes to the circular economy in the membrane technology field. Used RO membranes would otherwise end up in landfills, creating a huge burden operated at the pilot scale plant. Such R-ROs can be utilized by developing nations, and refined water can be obtained with a turbidity of <1 NTU, 100% removal of the total coliforms, and nitrate and pH being also under the prescribed WHO limits [48]. Detailed graphs of pH and nitrites can be found in Figures S1 and S2.

The sodium hypochlorite (NaOCl) exposure for 10 h also showed that the membrane remained consistent in its performance and can be used for drinking water purposes and for wastewater treatment polishing steps to further remove the bacterial contamination with the minimum resources utilization. The recycled RO membrane can be used in the replacement of the UF spiral wound membrane, which is the most economical and sustainable approach to reduce waste generation and recover the resource for the same usage. According to a study by Paula et al., replacing the UF spiral-wound membrane which has 5 years of life for water treatment with a recycled RO membrane with a life of 2 years provides 98.9% economic benefits [49]. Additionally, it is concluded that R-RO membranes have a potential to provide greater economic and environmental benefits while reducing waste [11,39,50].

This study proves recycled RO membrane’s effectiveness, performance on three sources of water, and resource recovery that can bring a lot of benefits to developing nations, if used membrane at a lower cost sells to them, instead of landing in landfills.

## 4. Conclusions

Previous research has been conducted on the conversion of recycled RO membranes to UF and being tested for different sources of feed as well. This study brings novelty by testing the converted R-RO membrane on the integrated constructed wetland permeate ICW-P and membrane bioreactor permeate MBR-P.

- Conversion of R-RO using the same concentration of NaOCl for 10 h depicted no variation in the results, and pH also remained constant.
- R-RO membranes proved to be very effective on TW, ICW-P, and MBR-P in terms of water recovery % that can be compared with the virgin UF spiral wound membrane.
- It also demonstrated an unprecedented turbidity removal percentage of 95% which is exactly equal to UF performance in some studies in the literature.
- The COD removal percentage was observed to be up to 67% using the R-RO membrane, which is in accordance with converted RO membranes in the literature.
- The highest number of total coliforms were present in all of the feed water from (TW, ICW-P, and MBR-P), but R-RO ensured it was safe for use by eliminating all of the total coliforms with a 95% confidence level.
- It proved to be an economically viable, environmentally friendly, and sustainable approach to convert the RO-used membranes and utilize them for water treatment of these origins because its product water is under the drinking water limits for TW and effluent discharge limits for ICW-P and MBR-P of the WHO and Pakistan’s NSDWQ (National Standards for Drinking Water Quality) and NEQS (National Environmental Quality Standards), respectively.
- Developing countries can leverage this by importing the used RO membrane from the developed nations and play a pivotal role in the reduction of waste and transition towards the circular economy.

## Figures and Tables

**Table 1:** RO membrane specifications and operating conditions.

Membrane	Model	Size (Inches)	Active Area ft^2^ (m^2^)	Maximum Operating Pressure	Maximum Operating Temperature	pH
Filmtec-BWRO	LC-LE-4040	40	94 (8.7)	(41 bars) 600 psig	113 °F (45 °C)	2–11

**Table 2:** Conversion procedure of spent RO and R-RO.

Runs	Membrane	Chemical Used	Exposure Time (h)	Method (AS or PS)	Dose Level (ppm/h)	Total Exposure Time (h)
2	Filmtec- BWRO (LC-LE-4040)	6% NaOCl (12.5% conc. aqueous solution)	5 h	Active System (CIP pump used)	300,000	5

**Table 3:** Initial physicochemical and microbial analysis of TW, ICW-P, and MBR-P.

	Water Type		
Parameters	TW	ICW-P	MBR-P
pH	7	8	8
EC (uS/cm)	1047	1614	1143
Turbidity (NTU)	1	25	1
COD (mg/L)	49	53	55
MPN (CFU/100 mL)	>23	>23	>23

**Table 4:** SEM-EDX number and percentage of elements present on the R-RO membrane surface.

Element	Weight %	Atomic %
C K	38.21	43.51
N K	37.24	36.36
O K	22.21	18.99
Na K	0.87	0.52
Al K	0.19	0.10
Si K	0.07	0.03
S K	0.85	0.36
Cl K	0.33	0.13
Ca K	0.02	0.01
	100.00	

**Table 5:** MPN results (CFU/100 mL) and removal of total coliforms from MPN index/100 mL.

	Intermittent Stages			
TW, ICW-P and MBR-P	Feed	CF	Recycled RO	UV
MPN (CFU/100 mL)	>23	>23	0	0
95% Confidence Limits	13	13	1–3.4	1–3.4
Removal %			100%	100%

**Table 6:** Product water values after R-RO and their comparison with standard limits.

	Water Type			Standards Limits		
Parameters	TW	ICW-P	MBR-P	WHO Standards	NSDWQ Pakistan	NEQS for Municipal and Liquid Industrial Effluent
pH	7	7.3	7.8	6.5–8.5	6.5–8.5	6–9
EC (uS/cm)	1035	1602	1143			
Turbidity (NTU)	0.8	0.8	0.6	<5 NTU	<5 NTU	
COD (mg/L)	49	53	55			150 mg/L
MPN (CFU/100 mL)	Not detected	Not detected	Not detected	Must not be detectable in 100 mL of water	Must not be detectable in 100 mL of water	
Nitrate (mg/L)	0.733	3.02	2.31	50 mg/L	≤50 mg/L	

## Data Availability

The data presented in this study are available on request from the corresponding author. The data are not publicly available due to maintaining and limiting the research to the trustable and reliable research journals only.

## Supplementary Materials

The following supporting information can be downloaded at: https://www.mdpi.com/article/10.3390/membranes13070628/s1, Figure S1: Average pH removal efficiency in TW, ICW-P and MBR-P using R-RO membrane (mentioned under heading of pH results in the supplementary materials document), Figure S2: Average nitrate removal efficiency in TW, ICW-P and MBR-P using R-RO membrane (mentioned under heading of nitrate results in the supplementary materials document), Figure S3: SEM micrographs at 1000 magnification (a) sample one (b) sample two (c) sample three of R-RO (mentioned under heading of SEM analysis at different resolutions in the supplementary materials document), Figure S4: SEM micrographs at 2000 magnification (a) sample one (b) sample two (c) sample three of R-RO (mentioned under heading of SEM analysis at different resolutions in the supplementary materials document), Figure S5: SEM micrographs at 500 nm scale (a) sample one (b) sample two (c) sample three of R-RO (mentioned under heading of SEM analysis at different resolutions in the Supplementary Materials document). References [51,52,53] are listed in Supplementary Materials.
