# Supplementary material for: Performance Evaluation of UF Membranes Derived from Recycled RO Membrane, a Step towards Circular Economy in Desalination
Source: Membranes (Basel). 2023 Jun 28;13(7):628. doi: 10.3390/membranes13070628 (PMC10385512; doi:10.3390/membranes13070628)
Supplement: Supplementary file 1 [file membranes-13-00628-s001.zip › membranes-2433462-supplementary.pdf]

<u>**Supplementary Data**</u>

Performance Evaluation of UF Membranes Derived from Recycled RO Membrane, a Step towards Circular Economy in Desalination

Zia Ur Rehman [1], Hira Amjad [1,*], Sher Jamal Khan [1], Maria Yasmeen [1], Aftab Ahmad Khan [2,3,*] and Noman Khalid Khanzada [4]

1 Institute of Environmental Sciences and Engineering IESE, National University of Sciences and Technology, NUST, Islamabad 24090, Pakistan
2 Department of Civil and Environmental Engineering, Hanyang University, Seoul 04763, Republic of Korea
3 Creative Engineering Consultants, Peshawar 25100, Pakistan
4 School of Energy and Environment (SEE), City University of Hong Kong, Hong Kong SAR, China
* Correspondence: hamjad@iese.nust.edu.pk (H.A.); aftab123@hanyang.ac.kr (A.A.K.)

pH Results

In figure S1 the average of 3 runs showed that in the TW pH decreased from 7.2 to 7 after, in ICW-P from the 7.5 to 7.3 and 8 to 7.8 in the MBR-P which depicts that after exposure of chlorine that cause the degradation of polyamide layer. While the permeability has increased over the time around the 7-8 pH, while another study described that performance at the 7 pH has decreased and at the 4 pH as well (Donose et al., 2013).

While this filmtec membrane demonstrated consistent performance and remained stable after the application of 3000 ppm/h of sodium hypochlorite as (Xie et al., 2022) in his study found out similar trend of stable pH after the sodium hypochlorite (NaOCl) exposure of 2000 ppm/h with higher water recovery of 20 % and saltwater rejection of <1 was observed. So, the above result of pH affirms that there is no contamination found in the product water as well after passing through the R-RO membrane in all type of waters (TW, ICW-P, and MBR-P) and during all the runs.

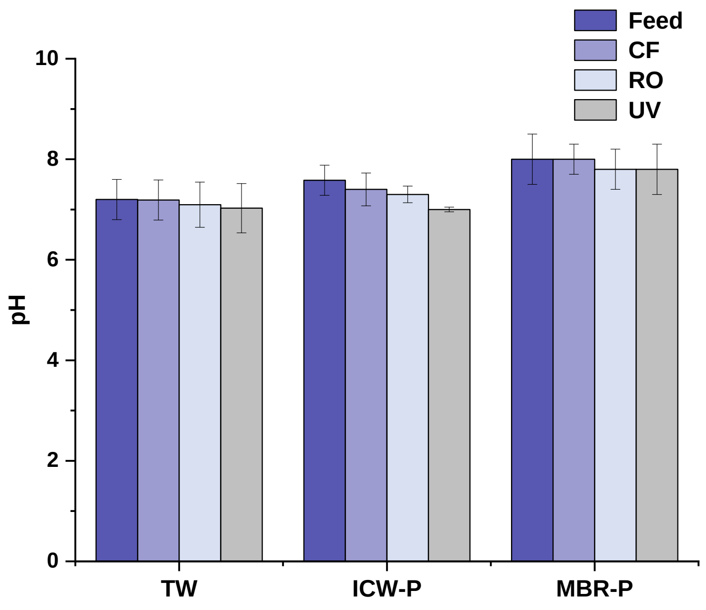

Figure S1. Average pH removal efficiency in TW, ICW-P and MBR-P using R-RO membrane

Nitrate Results

The average of 3 runs revealed that in the tap water nitrate slight decrease from 0.74 to 0.68 mg/l after the recycled RO, 3.4 to 2.9 mg/l in the ICW-P, and 2.48 to 2.40 mg/l in the MBR-P were observed as shown in the figure below.

All results of nitrate are under the WHO drinking water limits and under the national drinking water quality standards as well which poses no threat for its consumption and indicates that after passing through the R-RO the water. Applications of inorganic fertilizer and animal manure in agricultural regions are mostly to blame for the spike in nitrate levels in our water supplies in many parts of the world(Ward et al., 2018) . The current results show there's no such contamination in the feed water used in this study as well from all the 3 sources.

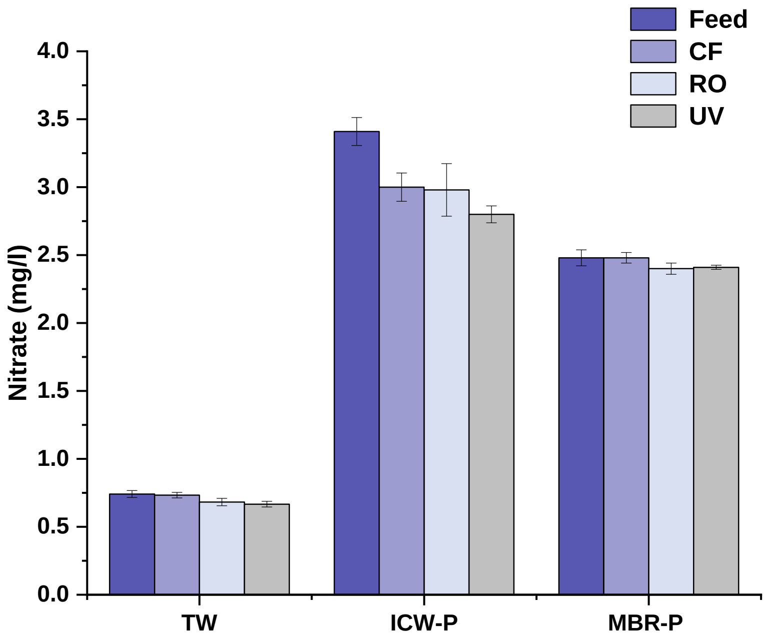

Figure S2. Average nitrate removal efficiency in TW, ICW-P and MBR-P using R-RO membrane

SEM analysis at different resolutions:

At the 1 µm the SEM analysis demonstrated the wide pores are present, some particles of large size and a rough surface morphology of the R-RO membrane can be observed in the sample a of figure S3. while in the sample 2 voids seem more in quantity but smaller in size and in case of sample 3 large size pores are evident. Also 2 large particles are present, the size shows maybe a small dust particle would have attached while handling.

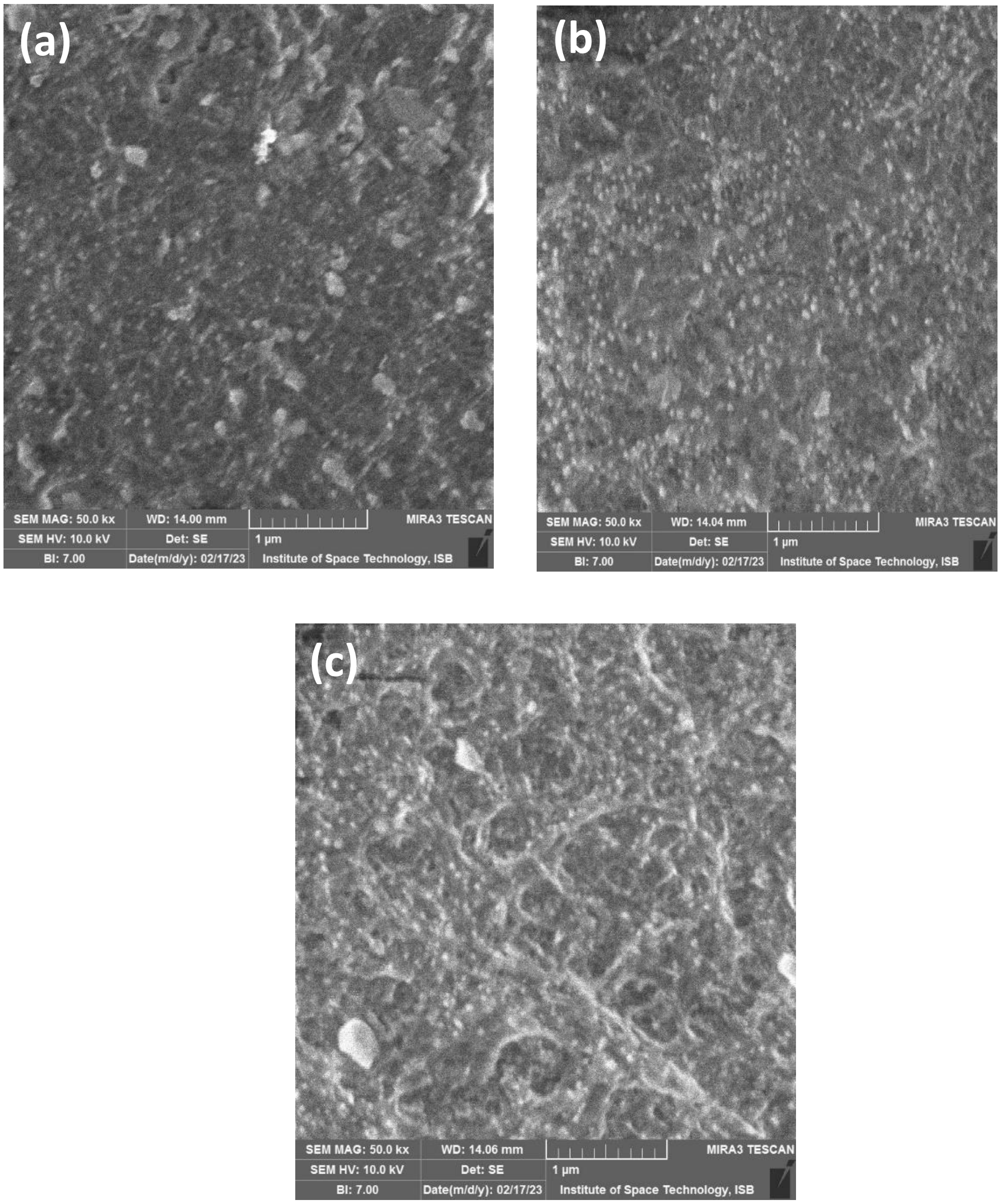

Figure S3. SEM micrographs at 1000 magnification a) sample one b) sample two c) sample three of R-RO

In figure S2. the micrographs at 2 µm were observed which shows that sample a has rough surface, with small size pores but now more crystal clear, while in case of sample 2 similar trend was observed irrespective of the tiny pores and less rough surface. It also reiterates the evidence of having pores size range from 44 nm to 71 nm, which certainly can be seen in these micrographs. At the 500 nm, the surface morphology shows evident pores in the sample one and likewise in the sample 2 and 3 in the below figure S3.

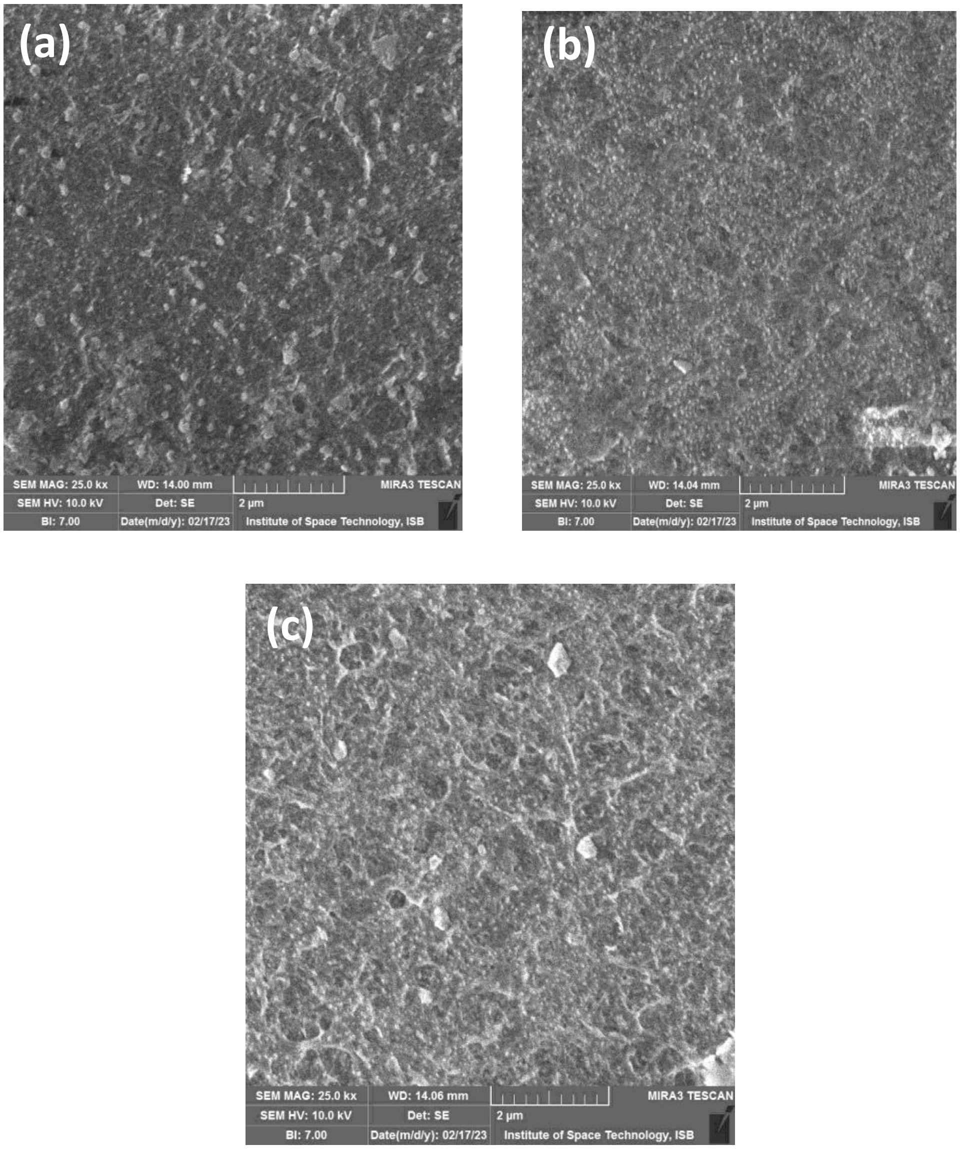

Figure S4. SEM micrographs at 2000 magnification a) sample one b) sample two c) sample three of R-RO

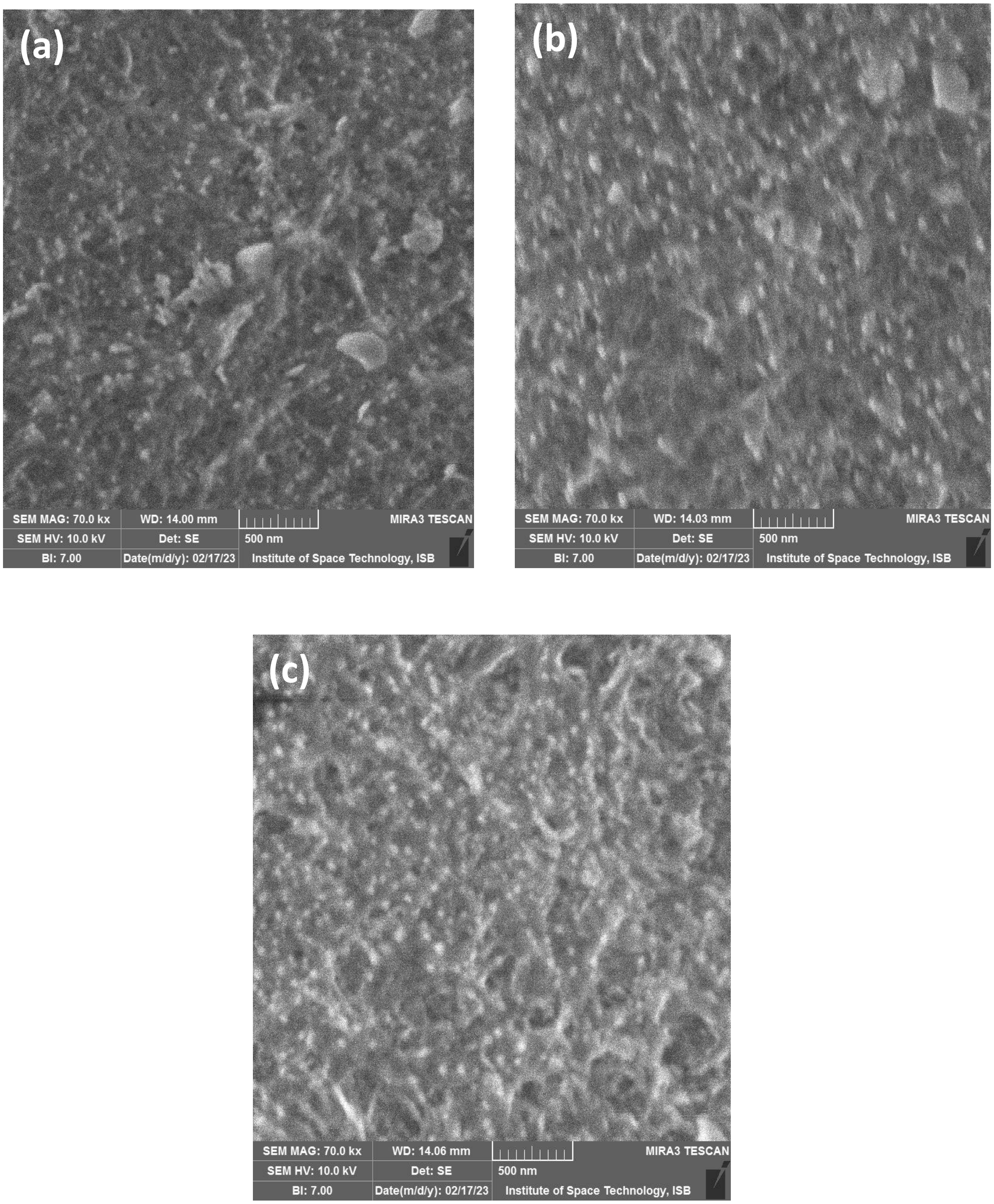

Figure S5. SEM micrographs at 500 nm scale a) sample one b) sample two c) sample three of R-RO

References

Donose, B. C., Sukumar, S., Pidou, M., Poussade, Y., Keller, J., & Gernjak, W. (2013). Effect of pH on the ageing of reverse osmosis membranes upon exposure to hypochlorite. *Desalination*, *309*, 97–105. https://doi.org/10.1016/J.DESAL.2012.09.027

Ward, M. H., Jones, R. R., Brender, J. D., de Kok, T. M., Weyer, P. J., Nolan, B. T., Villanueva, C. M., & van Breda, S. G. (2018). Drinking Water Nitrate and Human Health: An Updated Review. *International Journal of Environmental Research and Public Health 2018, Vol. 15, Page 1557*, *15*(7), 1557. https://doi.org/10.3390/IJERPH15071557

Xie, L., He, X., Liu, Y., Cao, C., & Zhang, W. (2022). Treatment of reverse osmosis membrane by sodium hypochlorite and alcohols for enhanced performance using the swelling-fastening effect. *Chemosphere*, *292*, 133444. https://doi.org/10.1016/J.CHEMOSPHERE.2021.133444
